# Acute Pain Service Utilization as a Lens on Inequities in Trauma and Inpatient Management

**DOI:** 10.3390/healthcare13233094

**Published:** 2025-11-27

**Authors:** Maxwell B. Baker, Rachel Achu-Lopes, Haley Mullins, Dhanesh D. Binda, Erin Dienes, Rose Joachim, Nicole Z. Spence

**Affiliations:** 1 Department of Anesthesiology, Boston University Chobanian & Avedisian School of Medicine, Boston, MA 02118, USA; rachel.achu@bmc.org (R.A.-L.); hmullins@bu.edu (H.M.); ddb96@bu.edu (D.D.B.); erin.dienes@bmc.org (E.D.); rcjoachim@gmail.com (R.J.); nicole.spence@bmc.org (N.Z.S.); 2 Larner College of Medicine, University of Vermont, Burlington, VT 05405, USA; 3 Department of Anesthesiology, Montefiore Einstein Medical Center, Bronx, NY 10467, USA

**Keywords:** acute pain service, inpatient pain management, health equity, safety-net hospital, trauma, substance use disorder, disparities

## Abstract

**Background:** Inequities in pain management are well documented in chronic pain and outpatient settings, yet little is known about disparities in inpatient Acute Pain Service (APS) care. This study evaluated demographic, clinical, and social factors associated with APS utilization and outcomes in an urban safety-net hospital, with a subgroup analysis of trauma patients who presented with at least three rib fractures. **Methods:** We performed a retrospective cohort study of two patient populations from our institution: (1) all patients receiving APS consultation from 1 January 2020 to 1 November 2022 (*n* = 1445) and (2) all patients with traumatic rib fractures during this time, stratified by APS consult status (*n* = 650). Demographics, insurance, comorbidities, opioid prescribing, and discharge outcomes were analyzed using descriptive statistics, multivariable logistic regression, and log-linear models. As APS consultation criteria were not standardized during the study period, referral patterns reflected routine clinical practice rather than predefined eligibility criteria. **Results:** Across the full APS cohort, patients were disproportionately represented from vulnerable groups: 30.5% were Black, 81.0% had public insurance, and 32.9% had a substance use disorder (SUD). Methadone use was a strong predictor of non-home discharge, including discharges to a medical facility, hospice, or against medical advice (AMA). In the rib fracture cohort, patients receiving APS consults had significantly higher injury severity scores (Injury Severity Score 17.1 vs. 13.0, *p* < 0.001). Black patients were less likely to receive APS consult (17.3% vs. 28.8%, *p* = 0.024). However, this association appeared to be attributable to the younger age and male predominance within this subgroup, as both factors were identified as significant predictors of APS consultation. **Conclusions**: APS utilization at a high-volume safety-net hospital highlights the intersection of medical vulnerability and structural inequities, with greater involvement among patients who were members of racial and ethnic minorities, publicly insured, or diagnosed with SUD. In trauma populations, younger Black men were over-represented, reinforcing the heightened injury risks Black men may face and the downstream effects on patient care. Taken together, these results highlight how APS involvement acts not only as a marker of increased injury severity but also as an intervention to improve care for vulnerable patient populations. As APS teams regularly serve these populations, they are well-positioned to bridge broader gaps through the integration of addiction and social support services, individualized pain management, and seamless coordination of care across specialties. These findings underscore the need for standardized consultation criteria and integration of social and addiction medicine resources into APS care models.

## 1. Introduction

Effective acute pain management is a cornerstone of high-quality care, particularly in urban trauma centers where emergency department (ED) overcrowding and limited resources are common. While an Acute Pain Service (APS) typically does not generate direct revenue through traditional billing methods, an APS offers substantial cost-saving benefits by mitigating opioid-related complications, shortening hospital stays, and preventing costly readmissions [1]. APS has markedly expanded over the past decade, with fellowship programs gaining accreditation from the Accreditation Council for Graduate Medical Education (ACGME) in 2016 [2]. Now an essential component of enhanced recovery after surgery (ERAS) pathways, acute pain management has been shown to improve outcomes by reducing pain, minimizing postoperative complications such as nausea and ileus, and enhancing mobility—factors that accelerate recovery and return to daily activities [3,4,5]

While APS has been shown to reduce postoperative complications, shorten length of stay, and lower total cost of care, contributing to value-based reimbursement goals, APS program structures still vary considerably across hospitals [1]. While tertiary academic centers often have 24-h, anesthesiologist-led APS coverage, community and safety net institutions may lack the staffing or financial resources to offer comparable services. These discrepancies can translate into differences in which patients receive consultation, the timing of interventions, and the consistency of multimodal pain management across institutions. Safety-net hospitals, which care for a disproportionate share of publicly insured and other vulnerable patient populations [6], must frequently balance limited resources with the need to manage complex perioperative pain, making them valuable settings for studying how equity and access intersect with APS utilization.

Disparities in pain medicine are well documented in outpatient and chronic pain settings, where racial and ethnic minorities, patients with public insurance, and individuals with substance use disorders are less likely to receive adequate pain treatment [7,8,9]. These structural inequities may extend into inpatient APS utilization, but have yet to be thoroughly studied. APS structures are heterogeneous across institutions, with leadership ranging from anesthesiologists trained in acute pain or regional anesthesia to perioperative hospitalists, advanced practice providers, and nurse-led teams. While this flexibility allows APS to adapt to local needs, it can also increase inconsistency in referral patterns and clinical decision-making. A growing body of research is revealing how vulnerable patient populations are at risk for both undertreatment of pain and higher rates of opioid related adverse events (ORAE) [10]. Patients with substance use disorders (SUD), for example, may be perceived as challenging to manage. In comparison, patients with obesity have been found to experience altered pain perception and increased risks of opioid-related complications [11,12]. The combination of obesity and comorbid conditions, including obstructive sleep apnea, can make deliberate opioid administration even more important to prevent unwanted ORAEs such as respiratory arrest. These patient-level differences intersect with systemic factors, raising the possibility that APS referral itself may reflect broader structural inequities in healthcare delivery.

Despite the critical role of APS in shaping inpatient outcomes, little is known about the demographic, clinical, and social factors that influence APS utilization, particularly among urban safety-net hospitals, where vulnerable populations are disproportionately represented. The primary objective of this study was to evaluate factors associated with APS utilization and to determine how APS involvement relates to patient outcomes, with discharge disposition used as a marker of post-acute care complexity and vulnerability. The secondary objective was to examine a high-risk subgroup of trauma patients with three or more rib fractures and compare those who received APS consultation with those who did not, as well as to assess whether APS utilization differed by demographic or clinical characteristics and how APS involvement may influence patterns of care and disposition.

## 2. Methods

This retrospective cohort study was conducted at Boston Medical Center, an urban Level I trauma center and safety-net hospital. The study was reviewed and approved by the Boston University Boston Medical Center Institutional Review Board (H-43356). Two cohorts were analyzed between 1 January 2020 and 1 November 2022: an APS cohort consisting of all inpatients who received a consultation from the APS during the study period (*n* = 1445), and a rib fracture cohort, consisting of all patients presenting with three or more rib fractures during the same period, stratified by APS consult status (*n* = 650). Patients and their respective diagnoses were identified using ICD-10 diagnostic codes and confirmed by chart review. Patients with incomplete data on consultation status or outcomes were excluded from analysis. APS consultations at our institution are limited to hospitalized patients, including those in the emergency department and observation units. For trauma patients, referral criteria include three or more rib fractures and age ≥ 65, in which case an APS consult is automatically placed. Consults for all other patients are initiated at the discretion of the treating clinician.

### 2.1. Variables Collected

Demographic variables included age, sex, race, and ethnicity (self-reported from a distinct list within the electronic medical record). Clinical and social factors included insurance status, comorbidities, and Injury Severity Score (ISS) for trauma patients. Care-related variables consisted of opioid prescribing and APS involvement. Outcomes included discharge disposition (home, skilled nursing facility, rehabilitation facility, hospice, or AMA).

### 2.2. Statistical Analysis

Descriptive statistics were used to summarize patient demographics, clinical characteristics, and outcomes. Continuous variables were reported as means with standard deviations or medians with interquartile ranges, as appropriate. Categorical variables were reported as counts and percentages.

Comparisons between groups were performed using two-sample t-tests for continuous variables and chi-square, or Fisher’s exact tests, when expected or observed cell counts are small, for categorical variables. Multivariable logistic regression was used to identify predictors of APS consultation, and multinomial regression was used to identify predictors of non-home discharge. Log-linear models were additionally applied to assess associations between demographic, clinical, and social factors and outcomes. Forward stepwise regression was used to develop the final models, which were adjusted for age, sex, and other covariates identified as clinically relevant or statistically significant on univariable analysis, including specific opioids administered during hospitalization and injury severity score (ISS) in the trauma cohort. Although additional socioeconomic factors, such as education, employment, and social support, were not included, these variables are unlikely to alter the results substantially.

All analyses were conducted using R 4.4.0 (R Foundation for Statistical Computing, Vienna, Austria) with packages dplyr and nnet [13]. A two-tailed *p*-value < 0.05 was considered statistically significant.

## 3. Results

### 3.1. Study Populations and Baseline Characteristics

During the study period, 1445 patients received an APS consultation and follow-up, and 650 patients presented with three or more rib fractures. Within the rib fracture cohort, 132 (20.3%) received an APS consult.

### 3.2. APS Cohort Characteristics

Among the 1445 APS patients, 30.5% identified as Black, 81% had public insurance, and 32.9% had documented SUD. Patients identifying as White had the highest rates of SUD and had significantly higher rates of opioid use disorder (OUD) than any other race (*p* < 0.001). The mean hospital length of stay was 11.2 ± 17.3 days. Discharge outcomes included 69.0% home, 24.7% facility, 3.3% AMA, and 1.7% hospice. Nearly all APS patients (97.7%) received opioids, with over half (55.0%) receiving three or more opioid medications (Table 1).

### 3.3. Disposition Outcomes in APS Patients

In multinomial regression models, methadone use was a strong predictor of non-home discharge. Compared with patients not receiving methadone, those administered had a greater likelihood of being discharged to a medical facility (OR 2.97, 1.98–4.46, *p* < 0.001), AMA (OR 3.24, 1.62–6.47, *p* = 0.001), and hospice (OR 4.33, 1.28–14.7, *p* = 0.018). SUD independently predicted AMA discharge (OR 4.03, 1.99–8.16, *p* < 0.001). Older age increased the odds of facility discharge (OR 1.04, 1.03–1.05, *p* < 0.001) and hospice discharge (OR 1.08, 1.05–1.11, *p* < 0.001). A sensitivity analysis was conducted by repeating the multinomial model separately for patients who received nonsteroidal anti-inflammatory drugs (NSAIDs) during their hospital stay (46.6%) and for those who did not (53.4%). The direction and magnitude of the estimated coefficients were consistent across both subgroups. While some predictors lost statistical significance, this was attributable to reduced sample sizes within outcome categories. These findings highlight the intersection of APS care with addiction and social vulnerability (Table 2).

Among 650 patients with rib fractures, 132 (20.3%) received an APS consultation. Compared with those without APS involvement, consulted patients were older (58.5 vs. 52.3 years, *p* = 0.001), more often women (40.9% vs. 24.7%, *p* < 0.001), and had higher ISS (17.1 vs. 13.0, *p* < 0.001). On unadjusted analysis, Black patients were less likely to receive an APS consult (17.3% vs. 28.8%, *p* = 0.024); however, this disparity attenuated after adjusting for age and sex.

### 3.4. Predictors of APS Consultation in Rib Fracture Patients

In multivariable logistic regression, older age (OR 1.017, 1.006–1.028, *p* = 0.002), female sex (OR 1.81, 1.181–2.770, *p* = 0.006), and higher ISS (OR 1.057, 1.033–1.080, *p* < 0.001) independently predicted APS consultation. Race was not independently associated with consultation status after adjustment (Table 3).

## 4. Discussion

Our study highlights how APS utilization reflects the intersection of vulnerability and inequity within an urban safety-net hospital. These findings also align with established frameworks on Social Determinants of Health, which emphasize how systemic factors, including socioeconomic status, access to care, and implicit bias, shape patterns of healthcare utilization [9,14]. In a cohort of over 1400 patients, individuals were overwhelmingly represented by groups already known to face structural barriers in healthcare and perioperative medicine, including patients with public insurance, racial and ethnic minorities, and those with SUD [7,9,15]. These findings suggest that APS involvement often occurs at the crossroads of complex social, clinical, and economic factors rather than as an isolated response to pain severity. Similar patterns have been described in chronic and rural pain management, where geographic and resource disparities can limit access to multidisciplinary care [16]. These parallels suggest that inequities in pain care can manifest across both inpatient and community settings. Within our cohort, this was reflected in discharge outcomes, as patients receiving methadone or with documented SUD were more likely to experience non-home discharge or leave AMA. This finding likely reflects confounding by indication, as patients receiving methadone for SUD often face overlapping medical, psychological, and social challenges that can complicate discharge planning [17,18]. This association demonstrates the intersection of APS and addiction medicine in caring for vulnerable patient populations as well as the potential role of integrated care pathways for patients with complex needs.

In the subgroup of patients with rib fractures, APS consults were more frequent among older patients, women, and those with higher ISS. In contrast, a disparity in consult rates among Black patients was observed, which attenuated after adjusting for age and sex. Importantly, younger Black men were disproportionately represented among trauma patients with rib fractures overall, consistent with epidemiologic data showing elevated trauma burden in this population [8]. This observation underscores how inequities in trauma epidemiology shape downstream care pathways. While the adjusted analysis suggests race was not independently associated with APS consult status, the disproportionate representation of Black men in the trauma cohort highlights the ways structural inequities can manifest before a patient enters the hospital. These results suggest that disparities in trauma incidence may shape injury patterns and also access to specialized pain management.

Our results may help extend existing literature on inequities in pain care. Prior work has consistently documented disparities in analgesic prescribing for acute conditions such as fractures or appendicitis in pediatric populations, in which Black and Hispanic children were less likely to receive opioids compared to White children [19]. Similarly, patients with public insurance have been shown to face greater barriers in accessing multimodal chronic pain treatments, including physical therapy and interventional procedures [9]. Our findings suggest that even within an inpatient APS intended to optimize pain management, utilization patterns mirror structural inequities already well described in outpatient and chronic pain settings.

The implications for clinical practice are significant. Instead of viewing APS involvement solely as a marker of injury or disease severity, our results highlight APS consultations as occurring at the intersection of pain, addiction, and social vulnerability. This framing suggests an opportunity for APS to expand its role beyond technical expertise in analgesia to central actors in equity-focused perioperative care. Embedding addiction medicine and social work into APS workflows may address the needs of patients with SUD or socioeconomic disparities. At the same time, case management support could improve discharge planning for those at risk of non-home dispositions. Standardized consultation criteria for inpatients, such as high-risk trauma patients with rib fractures, SUD, or methadone therapy, may reduce discretionary variation and ensure equitable access to multimodal pain care.

**Figure 1:**
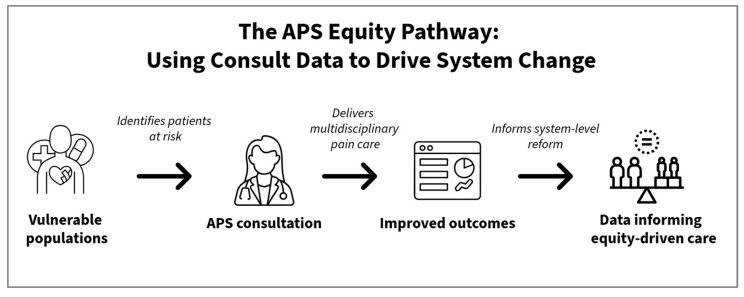
The APS Equity Pathway.

At a systems level, APS teams can also be leveraged as a critical cost-saving intervention (Figure 1). By reducing complications, preventing readmissions, and shortening length of stay, APS can demonstrate value even in resource-constrained settings [1,20]. When directed toward vulnerable populations, this value is social as well as economic, aligning with health equity goals increasingly emphasized in health policy. As the landscape of acute pain medicine evolves—with growing fellowship training, enhanced recovery after surgery protocol (ERAS) integration, and recognition of patient-reported outcomes—there is an opportunity to redefine APS not only as a consultative service but also as a structural tool for mitigating inequities.

Ultimately, APS utilization may serve a dual and critical role: as a marker of medical vulnerability and as an opportunity for targeted intervention. Patients receiving APS consults are often those most at risk of adverse outcomes, yet these very touchpoints may be leveraged to deliver more comprehensive, equity-oriented care.

### Limitations and Future Directions

This study has several limitations. Conducted at a single urban safety-net hospital, our findings may not generalize to other institutions with different patient demographics or APS structures. The retrospective design relies on EMR data, which is prone to coding errors, incomplete documentation, and misclassification of variables such as substance use disorders or opioid prescribing. Although injury severity was adjusted for using ISS, this metric may not fully capture the complexity of injuries or pain burden, and residual confounding remains possible. APS consultation patterns may also reflect discretionary factors, such as clinician bias, variable staffing, or consultation triggers, that were not measurable in our dataset. The study period overlapped with the COVID-19 pandemic, which affected hospital operations, admission patterns, consult availability, and discharge pathways, although these potential effects were not directly analyzed. While discharge outcome was selected as the primary outcome to reflect post-acute complexity and recovery needs, detailed longitudinal outcomes, such as readmission, ICU length of stay, or specific complications, were not consistently available for all patients. Finally, the small size of certain subgroups limits statistical power and precision of effect estimates.

Another limitation of this study was pain assessment: pain severity after APS consultation was discussed among care teams, but not consistently recorded using standardized Numeric Rating Scale (NRS) scores. Pain documentation can vary by provider, limiting the ability to objectively quantify pain improvement. As a result, discharge disposition reflected a combination of factors, including pain, overlapping medical, psychological, and social challenges. However, it cannot be explicitly concluded that any specific pain score predicted a non-home discharge. However, when taking the combination of these factors into consideration, a non-home discharge is still deemed a marker of vulnerability.

Similarly, while patients meeting specific criteria automatically received an APS consult, additional consults were initiated at the discretion of the treating provider. As a result, patients who did and did not receive an APS consult may have differed in terms of pain complexity or severity, introducing potential residual confounding. Given that NRS pain scores were not systematically compared between groups, it is difficult to determine whether differences in APS utilization reflected true variations in pain burden or provider judgment.

Future research should expand on these findings through multi-institutional and prospective studies that evaluate APS utilization across diverse health systems. Such studies would benefit from incorporating longitudinal and patient-reported outcomes to better capture the impact of APS on hospital recovery, discharge patterns, opioid use, and social reintegration. Qualitative research may further elucidate clinician decision-making, referral biases, and patient experiences not visible in quantitative data.

Interventional work is also needed. Standardized consultation criteria for high-risk populations may reduce discretionary variation, while embedding addiction medicine and social work into APS workflows could strengthen care continuity and potentially improve post-hospital discharge outcomes, such as reducing relapse and rehospitalization. Ultimately, future investigations can inform the development of evidence-based policies and multidisciplinary models of APS care that optimize pain outcomes while explicitly addressing inequities in referral and delivery.

## 5. Conclusions

APS utilization at our safety net hospital disproportionately involved vulnerable patients and reflects broader inequities in trauma epidemiology and pain management. Younger Black men were overrepresented among rib fracture patients, while methadone use and SUD predicted complex discharge patterns. As APS clinicians work with vulnerable patient populations who may require specialized care, they are uniquely positioned as consultants for pain management and as key stakeholders in equity-focused, multidisciplinary care for patients at the crossroads of trauma, addiction, and social vulnerability.

These results position APS not only as a consultative service for clinical benefit, but as a system-level mechanism for identifying and addressing disparities in inpatient pain management. As APS clinicians often interact with patients who may experience comorbidities complicating their pain management, such as social stressors and stigma, the role of APS extends beyond providing sufficient analgesic care. By engaging with psychiatry, addiction medicine, and social work, APS teams can bridge gaps between acute care and longitudinal recovery, promoting continuity of care for those who may be marginalized in traditional health systems.

In addition to its clinical and ethical importance, equitable APS aligns with emerging national priorities in value-based care [20].

## Figures and Tables

**Table 1:** Baseline characteristics of patients engaging with the Acute Pain Service, represented as mean ± standard deviation or count (%).

Baseline Characteristics	All Patients N (%)
**Total**	N *=* 1445
Age at date of visit (Range)	53.1 ± 16.8 (13–100)
BMI at visit (Range)	29.2 ± 7.6 (13–81)
Female Sex	791 (54.7)
Race	
American Indian/Alaska Native	9 (0.6)
Asian	46 (3.2)
Black/African American	441 (30.5)
Hispanic	87 (6.0)
Native Hawaiian/Pacific Islander	1 (0.0)
White	666 (46.1)
Other	143 (9.9)
Unknown	52 (3.6)
**Hispanic or Latino**	
Yes	257 (17.8)
No	1162 (80.4)
Unknown	26 (1.8)
**Primary Language**	
English	1198 (82.9)
Not English	239 (16.5)
Unknown	8 (0.6)
**Insurance Category**	
Public	1065 (73.7)
Private	210 (14.5)
Other	39 (2.7)
Unknown	131 (9.1)
**Insurance Subcategory**	
Accident/Liability	34 (2.4)
CommChoice/QHP	45 (3.1)
Commercial	165 (11.4)
Dual/SCO/PACE	125 (8.7)
Medicaid	566 (39.2)
Medicare	372 (25.7)
Unknown	138 (9.6)
**Comorbidities**	
Obesity	350 (24.2)
Depression	286 (19.8)
Anxiety	317 (21.9)
Fibromyalgia	32 (2.2)
Sickle Cell Anemia	21 (1.5)
CRPS	5 (0.3)
Chronic Back Pain	234 (16.2)
Substance Use	
Tobacco	250 (17.3)
Alcohol	150 (10.4)
Any Substance Use Disorder	476 (32.9)
**Number of Surgeries**	3.7 ± 3.5 (0–25)
**Opioids Administered**	
Methadone	164 (11.3)
Hydromorphone	1108 (76.7)
Morphine	367 (25.4)
Fentanyl	869 (60.1)
Oxycodone	1175 (81.3)
Any Opioid	1410 (97.6)
**Number of Opioids Administered**	
0	33 (2.3)
1	192 (13.3)
2	423 (29.3)
3	568 (39.3)
4	191 (13.2)
5	36 (2.5)
**Non-Opioid Pain Medication Administered**	
Any *	1439 (99.7)
NSAIDs Administered	673 (46.6)
**ED Disposition**	
Admit to inpatient	532 (74.4)
Against medical advice	1 (0.1)
Discharge	4 (0.6)
Admit to observation	178 (24.9)
**Discharge Disposition**	
Against medical advice	48 (3.3)
Deceased	17 (1.2)
Home	996 (69.0)
Hospice	25 (1.7)
Medical facility	357 (24.7)
**Length of Stay (Range)**	11.2 ± 17.3 (0.2–324.5)

* Any non-opioid pain medication includes acetaminophen; gabapentinoids (gabapentin, pregabalin); skeletal muscle relaxants (methocarbamol, cyclobenzaprine, tizanidine, carisoprodol); and NSAIDs (ibuprofen, naproxen, diclofenac, ketorolac).

**Table 2:** Multivariate-adjusted odds ratio of a patient being discharged to various locations versus being discharged home.

	Compared to Patients Discharged Home							
Variables	Medical Facility OR (95% CI)	*p*-Value (Adjusted)	AMA OR (95% CI)	*p*-Value (Adjusted)	Hospice OR (95% CI)	*p*-Value (Adjusted)	Deceased OR (95% CI)	*p*-Value (Adjusted)
Age	1.04 (1.03, 1.05)	<0.001 *	0.97 (0.95, 0.99)	0.005 *	1.08 (1.05, 1.11)	<0.001 *	1.03 (1.00, 1.06)	0.076
BMI	1.01 (0.99, 1.03)	0.194	0.93 (0.88, 0.98)	0.004 *	0.92 (0.86, 0.99)	0.030 *	1.02 (0.97, 1.08)	0.436
Female Sex	0.52 (0.40, 0.67)	<0.001 *	0.69 (0.37, 1.27)	0.233	0.34 (0.14, 0.83)	0.017 *	0.47 (0.17, 1.26)	0.134
Substance Use Disorder	1.12 (0.84, 1.48)	0.448	4.03 (1.99, 8.16)	<0.001 *	0.90 (0.33, 2.41)	0.829	0.99 (0.34, 2.90)	0.981
Methadone Administered	2.97 (1.98, 4.46)	<0.001 *	3.24 (1.62, 6.48)	0.001 *	4.33 (1.28, 14.7)	0.018 *	2.99 (0.75, 11.91)	0.121

* indicates the *p*-value is statistically significant at the 0.05 level.

**Table 3:** Multivariate-adjusted slope coefficients for predicting APS consultation.

Variables	Odds Ratio	95% CI	*p* (Adjusted)
Age	1.017	(1.006, 1.028)	0.002 *
Female Sex	1.813	(1.181, 2.770)	0.006 *
ISS	1.057	(1.034, 1.080)	<0.001 *

* indicates the *p*-value is statistically significant at the 0.05 level.

## Data Availability

Data is restricted for privacy reasons.
